# A double blind randomised control trial investigating the efficacy of platelet rich plasma versus placebo for the treatment of greater trochanteric pain syndrome (the HIPPO trial): a protocol for a randomised clinical trial

**DOI:** 10.1186/s13063-018-2907-x

**Published:** 2018-09-21

**Authors:** Eshan Oderuth, Mohammed Ali, Ismael Atchia, Ajay Malviya

**Affiliations:** 1 Chesterfield Royal Hospital, Chesterfield, UK; 2 Northumbria Healthcare NHS Foundation Trust, North Shields, UK

**Keywords:** Greater trochanteric pain syndrome, Trochanteric bursitis, Gluteus medius tendinopathy, Platelet-rich plasma

## Abstract

**Background:**

Greater trochanteric pain syndrome (GTPS) is a painful condition characterised by pain around the greater trochanter usually affecting middle-aged women. The majority of patients will improve with conservative management such as physiotherapy and non-steroidal anti-inflammatory drugs (NSAIDs); however, if this fails then more invasive treatments including corticosteroid injections and surgery may be required. Platelet-rich plasma (PRP) is an autologous blood product, which has a higher concentration of growth factors postulated to provide enhanced healing and anti-inflammatory properties. There have been numerous studies on PRP’s efficacy in musculoskeletal soft tissue conditions with similar pathology to GTPS, with varying results, most promising being in plantar fasciopathy and patellar tendinopathy. Corticosteroids are the established second-line treatment, but do not always work long term. PRP may be a suitable alternative to corticosteroid in GTPS with longer-term effects; however, there are very limited reports. The Hip Injections PRP Vs Placebo (HIPPO) trial aims to assess the ability of PRP to improve symptoms and function in patients with GTPS.

**Methods/design:**

HIPPO is a single-centre, double-blind randomized placebo-controlled study in patients with a radiologically confirmed diagnosis of gluteus medius or minimus tendinopathy with swelling within the tendon insertion with or without bursitis. We aim to randomise 102 patients with GTPS to either the PRP or placebo (normal saline injection) treatment arm. Participants will receive one ultrasound (US) guided PRP/placebo injection into the trochanteric bursa and surrounding gluteus medius/minimus tendons. The primary outcome measure is the International Hip Outcome Tool-12. Secondary outcome measures will include a visual analogue score for pain, the three-level version of the EuroQol five-dimensional questionnaire and the modified Harris Hip Score. Outcomes will be measured at baseline, 3, 6 and 12 months. Participants will have the option at 6 months to have a repeat blinded injection or cross over to PRP. Analyses of primary and secondary outcomes will be made according to the intention-to-treat principle. The trial reporting will comply with Consolidated Standards of Reporting Trials (CONSORT) guidelines.

**Discussion:**

The HIPPO study has been designed to test the hypothesis that PRP is effective in treating GTPS in patients who have failed conservative management and to assess the duration of effect of PRP.

**Trial registration:**

ClinicalTrials.gov, NCT03479190. Registered on 27 March 2018.

**Electronic supplementary material:**

The online version of this article (10.1186/s13063-018-2907-x) contains supplementary material, which is available to authorized users.

## Background

### Overview

Greater trochanteric pain syndrome (GTPS), also known as trochanteric bursitis, can be a debilitating condition characterised by pain around the greater trochanter. Pain increases on walking, squatting or climbing stairs and when lying on the affected side or when crossing one’s legs [1–3]. GTPS was a term adopted by Karpinski et al. in 1985 instead of trochanteric bursitis as patients did not exhibit typical bursitis signs of swelling, heat, crepitus or fluctuation [4]. This notion has been supported by other studies [5] including a study performed by Bird et al. 2001. They evaluated 24 patients using magnetic resonance imaging with the clinical picture of GTPS and found that the majority of patients had gluteus medius tendon pathology with only 2 patients with isolated trochanteric bursal inflammation [1]. Hence, GTPS instead of trochanteric bursitis appears to be the more accurate way of describing the clinical condition, which clearly may have multiple facets in its pathology.

A selection of non-surgical management options is normally recommended, including rest, ice, stretching, heat, strengthening and oral anti-inflammatory drugs. The majority of patients improve with conservative management; however, if this fails then more invasive treatment options may be explored, including shockwave therapy, corticosteroid injections [6–8] and even surgery. Corticosteroid injection is an established second-line treatment for GTPS that has been shown to be efficacious but not necessarily in the long term, as reported by previously published studies [9]. This notion has been supported by numerous reviews of GTPS management [6, 10–12]. Surgery is usually set aside until the condition has become refractory and conservative measures have been exhausted. Reports of PRP use as a novel therapy in GTPS are very limited. A review of treatments for GTPS by Buono et al. stated the following: “as platelet rich plasma (PRP) has been widely used to stimulate biological tendon healing in patients with chronic tendinopathies, futures trends of research and trials should focus on the application and effectiveness of PRP for the management of GTPS” [11].

### Existing knowledge

PRP is an autologous blood product, which has been postulated to promote healing in damaged or inflamed tissues including muscles, ligaments, bones and tendons. Platelets contain a variety of elements including growth factors and cytokines, which are involved in tissue healing. These include, platelet-derived endothelial growth factor (PD-EGF), platelet-derived growth factor (PDGF), transforming growth factor (TGF), insulin-like growth factor (IGF), vascular endothelial growth factor (VEGF) and basic fibroblast growth factor (bFGF). These growth factors are present in the processes of tissue injury, inflammation and repair. Therefore, theoretically the higher the concentration of platelets, the more growth factors there will be present to promote the healing process when administered directly to the area of interest [13].

PRP has been applied in other fields of medicine including regenerative therapies in oral and maxillofacial surgery [14, 15] Over the past decade or so there have been numerous studies of the efficacy of PRP in treating musculoskeletal conditions similar in pathology to GTPS, such as lateral epicondylitis, patellar tendinitis, rotator cuff pathology, Achilles tendinopathy and plantar fasciopathy. This has been collectively reviewed by several authors with reports of mixed efficacy compared to standard treatments for these conditions with the most promise shown in plantar fasciopathy and patellar tendonitis [16–18]. These reviews all mention the lack of evidence to fully support or reject the efficacy of PRP in these conditions, except for Achilles tendinopathy where PRP showed no difference compared with placebo in a randomised study [19, 20]. The healing or regenerative properties of PRP have shown promise in other orthopaedic areas such as cartilage pathology [21].

### Aims

The aim of the trial is to investigate the clinical efficacy of the novel treatment platelet-rich plasma (PRP) in treating patients with GTPS. The clinical efficacy of PRP will be compared against a placebo injection of normal saline.

### Hypothesis

PRP is an effective treatment for greater trochanteric pain syndrome.

### The need for the trial

GTPS can be a painful and debilitating condition, which as a last resort requires surgery especially if it does not respond to conservative treatment. This is clearly a developing field with a lack of published research with on-going studies investigating the efficacy of PRP in GTPS. Our trial differs from the other trials in its design as we will compare PRP with placebo. Our aim is to conduct a well-designed robust trial to establish whether PRP is effective compared to placebo and what the duration of the effect is for patients suffering from GTPS.

## Methods

### Trial design

The trial will be a two-arm, single-centre, double-blind, randomised control trial (RCT). The study will include a two-way comparison between PRP and placebo normal saline injections for treating GTPS. The trial design has been based on our local experience with using PRP in patients with GTPS and an RCT conducted by Monto et al. in 2014 [22] comparing steroid injection with PRP in patients with severe hip bursitis. Add to this, a pilot study by Ribeiro et al. in 2016 [23], which compared the efficacy of PRP against corticosteroid in the treatment of GTPS.

Participants will be identified and referred for inclusion in the study by their main care provider (general practitioner (GP), orthopaedic surgeon, rheumatologist or extended-scope physiotherapy practitioner). They will then be invited for a first interview where eligibility will be assessed, further information about the study will be given and written consent for inclusion obtained. Potential participants will be permitted to reschedule another interview appointment if they need more time to think about whether they wish to participate. Participants will be allocated randomly to either the PRP or normal saline injection treatment arm. They will receive their treatment under sterile conditions under ultrasound guidance by a consultant rheumatologist (IA). The participant will be contacted by phone a week after receiving treatment to monitor for early adverse events. Participants and outcome assessors will be blinded to treatment arm. Participants will be reviewed at baseline, 3, 6 and 12 months with patient-reported outcome measures (PROM) completed at each of these reviews. During the 6 months follow up participants will be given the option of a repeat injection of their original treatment or if they specifically request PRP then we will offer this to them but maintain the blinding of their original treatment. This was a key ethical discussion point in our focus group meeting engaging patient and public involvement in that expecting participants to potentially continue with placebo for a further 6 months while in pain would place a significant burden on them. The trial is expected to last approximately 4 years allowing 18–24 months for recruitment with the remaining time used to complete the follow up period until the last patient recruited.

The feasibility and scientific quality of the trial has been peer-reviewed and approved by the Principal Investigator (PI) and Research and Development team. The ethical approval was granted by Health Research Authority (HRA) England and the trial is listed with the number 198415. The trial protocol will permit its reporting in line with the Consolidated Standards of Reporting Trials (CONSORT) guidelines [24]. [The Standard Protocol Items: Recommendations for Interventional Trials (SPIRIT) checklist is provided as Additional file 1].

## Recruitment

The source of recruitment will be from their main care provider (GP, orthopaedic surgeon, rheumatologist or extended-scope practitioner). Following the first interview and consent to enter the trial, eligibility checks (Table 1) will be repeated for each participant on the day they attend for treatment, to ensure that participants are not randomised in error. The participant will receive confirmation of their inclusion in the trial, which will also be recorded in their medical notes. Their GP will be informed.

**Table 1:** Inclusion and exclusion criteria

Inclusion criteria:	
Over 18 years of age	
Symptoms consistent with GTPS present for at least 6 months	
Radiological diagnosis of GTPS using MRI, or ultrasound scan if MRI contraindicated	
Failed conservative management in any other care setting	
Patient is willing and able to provide written informed consent.	
Exclusion criteria:	
Lacks capacity to provide consent	
Has hip joint osteoarthritis demonstrated on a plain radiograph, requiring treatment	
Presence of confounding pathologies on the hip MRI	
Any extensive surgery or deformity of the hip demonstrated on x-ray	
Presence of systemic disorders – coagulopathy, active infection, immune system disorders, peripheral neuropathy, malignancy, unresolved fractures	
Had any surgical treatment specifically targeted at GTPS e.g. bursectomy/ilio-tibial band lengthening	
Pregnancy	
Anti-coagulant therapy e.g. warfarin, rivaroxaban, apixaban, dabigatran	
Haemaglobin < 10 g/dl or platelets < 150,000/ul	
Unable to safely stop anti-platelet/NSAID medications e.g. recent cardiac stenting	
Has lumbar-sacral spine pathology or a recent history of acute hip trauma	
Has a recent history of acute sciatica	
Is not able to attend or comply with treatment or follow up scheduling	
Participates in any other clinical trial	

### Consent

All potential participants will attend a first interview meeting with our research team following referral from their primary care provider (e.g. GP/orthopaedic surgeon/rheumatologist). They should already have received the trial information sheet. We will explain the purpose and nature of the trial again and assess their eligibility. They will be given up to a week to decide whether they wish to be entered into the trial. A second interview will be rescheduled if necessary. Written consent to enter the trial will be obtained. Once the participant has consented, their baseline PROMs will be assessed and recorded.

## Treatment allocation

### Sequence generation

The allocation sequence will be generated randomly using an online computer-generated randomiser (https://sealedenvelope.com/). The participant will be allocated to either the PRP or normal saline arm of the trial. All injections will be performed by the same consultant rheumatologist who will be not involved in the data collection process.

### Allocation concealment

The allocation sequence will be hidden from the PI and outcome assessors. Their allocation will be recorded on a separate database to which the PI/outcome assessors will not have access. Only the treatment administrator (consultant rheumatologist) will have access to this so they know what treatment they are issuing. They and a dedicated research nurse will be guardians of this allocation sequence database to ensure that the patients, PI and outcomes assessors do not have access. Allocation will be revealed once the trial has ended and data analysis begins.

### Allocation implementation

Participants will be enrolled by our research team lead by the PI (AM). They will have been allocated their treatment randomly. Allocations will be held on a secure database which the treatment administrator (consultant rheumatologist (IA)) will have access on the day of their treatment.

### Blinding

All participants will be blinded to the treatment allocation. All treatments will be prepared in another room and the patient will have a screen between them and their hip preventing them from seeing what treatment they are being given. The treatment administrator will not be blinded. Outcome assessors will be blinded.

### Trial treatments

All participants will attend the hospital as a day case. As per Northumbria Healthcare NHS Foundation Trust policies, written consent will be obtained for their procedure, which will be generic to cover both treatment arms. All participants will have 40 ml of blood drawn using an aseptic technique, which will then be taken to another room for 20 min to simulate the centrifuge time for the PRP preparation, regardless of which treatment they are receiving. They will also have a drape obscuring their hip and treatment area from their line of sight. All these measures are to maintain blinding of the participant to treatment allocation. The procedure will take place in sterile conditions to minimise the risk of infection. They will have local anaesthetic infiltrated superficially and deep in the greater trochanter area. Our consultant rheumatologist will then inject either PRP or normal saline under ultrasound guidance into the trochanteric bursa and abductor tendons (2 ml in the abnormal tendon and 2 ml in the bursa). All participants will then be advised to rest for 72 h and will be referred for physiotherapy.

Participants will be randomised into 2 groups:

1. Test: PRP treatment using the SW-PRP system provided by NTL Biologica – 40 ml of the participant’s own blood is added to 6 ml of ACD-A (Anticoagulant Citrate Dextrose Solution, Solution A). This is then placed into the SW-PRP Syringe Device. The vial is centrifuged at 3850 rpm for 7 min. Red blood cell fluid level in the device is optimised and then centrifuged for a further 4 min at 3850 rpm. Platelet-poor plasma is extracted from the device and discarded. The remaining PRP is extracted from the buffy coat layer; 4 ml of PRP is withdrawn and is ready for administration.
2. Placebo: 4 ml of normal 0.9% saline.

### Physiotherapy

The physiotherapy protocol will include stretching exercises, which consist of gluteal, iliotibial band and piriformis stretching. Prone lumbar extension, supine lumbar rotation and cat stretch exercises will also be included. These are accompanied with strengthening exercises such as side-lying leg lift, clam, gluteal kickback and balance exercises. This will be a general standardised physiotherapy regime and will be standardised across our physiotherapy requests. Naturally the physiotherapy that participants receive may vary, such as by the frequency of sessions, and we accept this as a variable that could influence outcome; however, measures will be taken to minimise this as much as possible.

### Measure of harm and adverse events

Participants will be monitored for adverse events at each follow up. One week after their treatment each participant will receive a phone call to monitor early adverse events. Adverse events will be reported in the study publication.

Expected potential adverse events:

- Pain
- Infection
- Failure to relieve/recurrence/progression of symptoms/exacerbation

## Outcome measures

### Primary

The International Hip Outcome Tool–12 (iHOT-12) PROM scores at baseline, 3, 6 and 12 months will be compared within and between the PRP and normal saline arms.

### Secondary

**Fig. 1:**
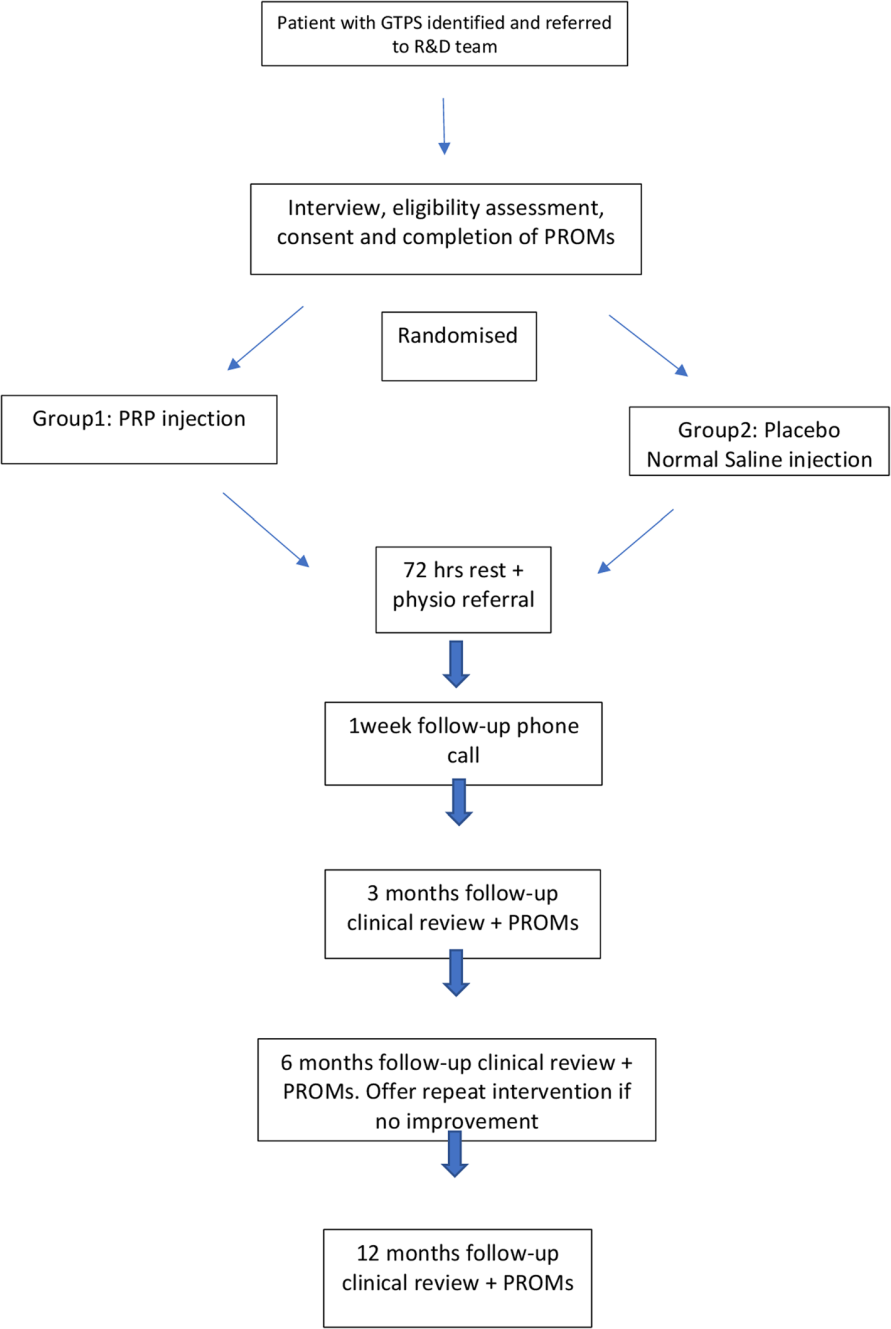
Standard protocol items: recommendation for interventional trials (SPIRIT) flow diagram. A summary of the planned study interventions and assessments. MRI, magnetic resonance imaging; iHOT-12, International Hip Outcome Tool-12; VAS, visual analogue score; EQ-5D 3 L, Three-level version of the EuroQol five-dimensional questionnaire; MHHS, Modified Harris Hip Score; PRP, platelet-rich plasma

The visual analogue score (VAS) for pain, the Modified Harris Hip Score (mHHS) and the three-level version of the EuroQol five-dimensional (EQ-5D 3 L) questionnaire PROM scores will be collected at baseline, 3, 6 and 12 months and will be compared within and between the PRP and normal saline arms (Fig. 1).

### End of the trial

**Fig. 2:**
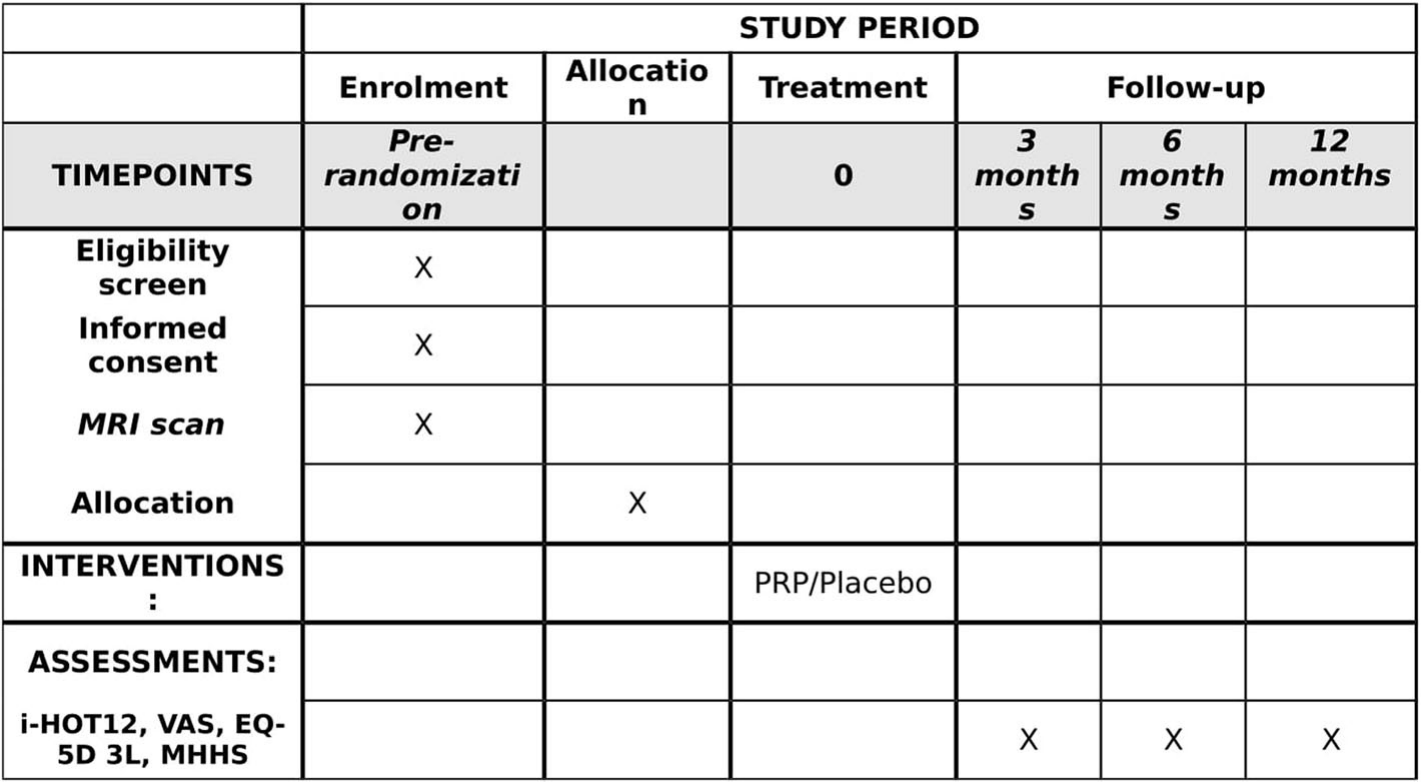
Study flow chart. GTPS, greater trochanteric pain syndrome; R&D, Research and Development; PROM, patient-reported outcome measure; PRP, platelet-rich plasma; physio, physiotherapy

Recruitment will cease once the last participant has been recruited following our sample size recommendations. The trial will cease once the last patient has attended their 12 months follow up (Fig. 2).

## Data management

The data collected from the trial will be entered into a trial database. The database will be agreed and set up by our information technology (IT) technician, statistician and PI. The database will be stored securely on our computer systems in the hospital. During the interim analysis, the database will be frozen to ensure data collected after this point are not included in the interim report. Access to the data will be limited to those only directly involved in the trial and who are part of the research team. The data will be anonymised in terms of participant identifiable data and will only contain demographic details. Identifiable participant data will be held on a separate database in our secure computer network in the hospital. Each participant will have a unique participant code so their outcome data can be matched with their personal identifiable data if required. All physical data will be stored in the Research and Development Office, North Tyneside General Hospital in a locked cabinet. The office is secured by a code-operated lock and is only accessible by the research team. All computer data will be stored on our secure password-protected National Health Service (NHS) Trust computers. Data will be archived in accordance with the Research and Development Department, Northumbria Healthcare NHS Foundation Trust guidance. A data monitoring committee will be formed and will convene for this trial.

### Data Monitoring Committee

A Data Monitoring Committee will convene at the interim period of 50% recruitment at 6-month follow up. This will be chaired by the PI and include other co-investigators, a trial coordinator and ideally a member of our patient focus group. All issues relating to the management and conduct of the trial will be reviewed and addressed. Meeting times will be an interim meeting at 50% recruitment reaching 6-month follow up and the end of trial.

### Statistical analysis

#### Statistician report

The primary outcome of interest is the change from baseline to 12-month follow up in the iHOT-12, comparing the PRP and placebo group. The study will be run as a superiority trial. The cut-off for statistical significance is set at 5% and desired power at 90%, with two-tailed tests applied. Data on change from baseline are also assumed to be parametric and the *t* test will be applied to the data to assess statistical significance.

The minimally clinically important difference (MCID) for the iHOT-12 has been reported by Sansone et al. as 10 (from 100) and the standard deviation for the change in score from baseline as no greater than 21 [25]. Although, there are few previous data on which to base a sample size calculation, Monto et al. compared change from baseline to 12-month follow up in a group of 40 patient with hip bursitis, with steroids and PRP as the interventions being compared. In the PRP group Harris Hip scores increased from 51.7 to 87.4 whilst in the steroid group, scores increased from 50.5 to 58.8 at 12 months [22]. Since the Harris Hip score is also scored from 100, our sample size calculation is based on these figures. We conservatively assume that change in iHOT score from baseline in the placebo group will be no more than in the steroid group reported by Monto et al., and estimate a maximal change of 10. We also estimate that the change in the iHOT score from baseline in the PRP group will be no less than 27. Using these figures, a minimal sample size at follow up of 66 (33 in each group) will be required.

Pilot data obtained by our team suggest that the rate of refusal to participate should be no more than 25% and the dropout rate no more than 35% over 12 months. Refusal rates tend to be low in this patient group given the chronic nature of the condition and the fact that patients will only be approached once conservative management has failed. Thus, we will need to approach 135.4 (rounded up to 136) patients, and recruit 102 patients to achieve our target sample size.

Data will be analysed using standard statistical software (e.g. SPSS and SAS). In the first instance, data will be analysed using simple descriptive statistics to compare the two groups in terms of demographics, clinical characteristics at baseline and outcomes. The primary outcome of interest will be the change from baseline to 12-month follow up in the iHOT-12, comparing the PRP and placebo group. The iHOT-12 data are expected to be parametric and so the unpaired *t* test will be used to compare the difference in change from baseline in the two groups. Since randomisation will not be stratified, we will also look to adjust for the possible confounding influence of differences in baseline characteristics (e.g. age, gender and body mass index (BMI)) using multivariable methods (e.g. linear regression). The significance level for all inferential tests will be set at 5%.

In secondary analysis, we will investigate changes in visual analogue pain score, EQ-5D 3 L and modified Harris Hip score in the two groups, as for the primary outcome. In sub-group analysis, we will investigate the data based on specific previous treatment, patients who required more than one treatment and patients with the highest levels of baseline pain.

The test group (PRP) alone: for all outcome measures the difference between the follow up scores with baseline will be assessed (e.g. baseline versus 3 months, baseline versus 12 months). The difference between one follow up period to the next will be examined (e.g. 3 months versus 6 months, 6 months versus 12 months). A significant difference will be set at a *p* value of less than 0.05.

The placebo group (normal saline) alone: data will be analysed as for the test group, as described.

Test versus placebo: for all outcome measures, the differential change at each follow up time point will be compared between the trial arms and the significance value calculated.

Statistical tests will be performed to ensure that gender, BMI and age differences are not significantly associated with a particular result. Care will be taken to minimise missing responses and to continue to follow up those who withdraw from treatment.

We will only be using the completed cases for final analyses; the remainder will be excluded.

## Discussion

Platelet-rich plasma (PRP) is an autologous blood product, which has a higher concentration of growth factors postulated to provide enhanced healing and anti-inflammatory properties. There have been numerous studies on the efficacy of PRP in musculoskeletal soft tissue conditions with similar pathology to GTPS, with varying results, the most promising being in plantar fasciopathy and patellar tendinopathy. Corticosteroids are the established second-line treatment, but do not always work long term. PRP may be a suitable alternative to corticosteroid in GTPS, with longer-term effects; however, there are very few reports discussing its efficacy. Lee et al. in 2016 [26] prospectively studied the efficacy of intra-tendinous PRP injections as treatment for chronic recalcitrant gluteus medius tendinopathy. They found ultrasound-guided intra-tendinous PRP injections to be a safe and effective treatment option for chronic recalcitrant gluteus medius tendinopathy due to moderate to severe tendinosis. A second report by Ribeiro et al. from Brazil [23] was published in 2016 comparing the efficacy of PRP against corticosteroids. They concluded that during the first 2 months, which was the study period, the PRP has no influence on pain relief and functional improvement in trochanteric syndrome. Again in 2016 a third report from the USA compared ultrasound-guided percutaneous tendon fenestration to PRP injection for treatment of GTPS [27]. The report concluded that both ultrasound-guided tendon fenestration and PRP injection are effective for treatment of gluteal tendinosis, showing symptom improvement in both treatment groups. Large effect sizes were evident in these studies. We calculated these based on Cohen’s test, taking some statistical assumptions into account, given that all data were not available. The effect sizes in the study of Lee et al. were 1.303, 1.052, 0.883 and 1.677 for the mHHS, Hip Outcome Score (HOS) activities of daily living, HOS sport and HOT-33, respectively. The effect sizes in the study of Ribeiro et al. were 1.155, 1.371 and 1.509 for the Facial Expressions Scale for Pain at follow up of 10, 30 and 60 days.

Prior to these reports, Mautner et al. conducted a study in 2013 [28] to assess whether ultrasound-guided PRP injections are an effective treatment for chronic tendinopathies. The study included 180 patients of whom16 had gluteus medius tendinopathy. The majority experienced significant improvement in symptoms, evidenced by 82% of patients reporting at least moderate improvement of their symptoms. A similar satisfaction rate of 85%was also found, respectively, demonstrating that the improvement in symptoms likely resulted in patient satisfaction. There are no randomised trials in the current literature and all these published reports were based on short follow up periods and small numbers of patients.

This paper describes the rationale and design for the first randomised trial that aims to determine the effectiveness of PRP in the treatment of greater trochanteric pain syndrome. No previous trials have assessed PRP against placebo or measured clinical outcomes beyond 6 months. The HIPPO trial will make a significant contribution to the evidence base available to support effective conservative management of GTPS.

### Current status of the trial

The trial is currently running. Recruitment and treatment started in spring 2018.

## Additional file

Additional file 1: Standard protocol items: recommendation for interventional trials (SPIRIT) 2013 checklist. Recommended items to address in a clinical trial protocol and related documents. (DOC 121 kb)

## Abbreviations

bFGF: Basic fibroblast growth factor
BMI: Body mass index
CONSORT: Consolidated Standards of Reporting Trials
EQ-5D 3 L: Three-level version of the EuroQol five-dimensional questionnaire
GP: General practitioner
GTPS: Greater trochanteric pain syndrome
IGF: Insulin-like growth factor
iHOT-12: International Hip Outcome Tool-12
mHHS: Modified Harris Hip Score
NHS: National Health Service
NSAID: Non-steroidal anti-inflammatory drug
PD-EGF: Platelet-derived endothelial growth factor
PDGF: Platelet-derived growth factor
PI: Principal Investigator
PROM: Patient-reported outcome measures
PRP: Platelet-rich plasma
RCT: Randomised control trial
TGF: Transforming growth factor
VAS: Visual analogue score for pain
VEGF: Vascular endothelial growth factor
